# Getting back to business: considerations for restarting non-cancer gynaecological surgery following the COVID-19 peak

**Published:** 2020-08-05

**Authors:** F Odejinmi, TJ Clark, R Mallick

**Affiliations:** Whipps Cross Hospital, Barts Health NHS Trust, Whipps Cross Road, Leytonstone, London, United Kingdom;; Department of Obstetrics and Gynaecology, Birmingham Women’s and Children’s Hospital, Birmingham, United Kingdom;; Princess Royal Hospital, Brighton and Sussex University Hospitals NHS Trust, Lewes Road, Haywards Heath, United Kingdom.

**Keywords:** COVID-19, coronavirus, surgery, laparoscopy

## Abstract

As we begin to pass the first peak of the coronavirus pandemic, the backlog of routine gynaecological surgical work is becoming more apparent and continues to build day by day. The potential for further pandemic surges remain; however it is imperative that elective gynaecological surgery is restored safely, ethically and in a timely manner. The risks of COVID-19 transmission and potential increased surgical morbidity must be weighed up against the patient’s ongoing symptoms and quality of life. Universal screening and testing of patients attending for routine surgery, as well as staff testing and retesting, will be fundamental to reducing the risks to both patients and staff, and avoiding the higher morbidity encountered when operating on asymptomatic infected patients. The aim of this paper is to explore pathways to safely reintroduce elective benign gynaecological surgery and the challenges that will be encountered including patient counselling and informed consent, surgical prioritisation and the screening and testing of patients and staff, as well as the logistical and ethical challenges of reintroducing benign surgery during COVID-19 times.

## Background

On the 11 th March 2020, the World Health Organization (WHO) declared a global pandemic caused by the very contagious SARS-CoV-2, the virus responsible for COVID-19 and a member of the β coronaviruses (CoVs), which first emerged in China in late 2019. It was quickly realised that left unchecked the virus would overwhelm society and its healthcare delivery systems.

Drastic measures were imposed to control the spread of the virus worldwide, including social distancing, enhanced hand washing hygiene to exploit the vulnerabilities of the virus, locking down society to curtail airborne and fomite viral spread. and a nationwide re-distribution of healthcare services. This included an expansion of intensive care capacity as well as a reduction in all non-essential elective work in order to cope with the resource diversion and “flatten the curve”. This has led to the widespread deferral of elective sur- gery with only cancer and urgent work allowed to continue, a move fully endorsed by the Royal Colleges and other national and international bodies (British Society for Gynaecological Endoscopy, 2020; European Society for Gynaecological Endoscopy, 2020; American Association of Gynecologic Laparoscopists, 2020a; Saridogan and Grimbizis 2020).

With the increasing backlog and potential for further waves, it is imperative that elective surgery is reintroduced safely and ethically once the first wave of the pandemic is over.

The aim of this paper is to review the current evidence and assess challenges that will be faced, and potential pitfalls that may be encountered when attempting to restart “normal” gynaecology services in COVID-19 impacted times. The only constant in this pandemic is the fact that with the passage of time our knowledge of SARS-CoV-2 will increase, and that guidelines will continue to change. In order to safely reintroduce benign gynaecological surgery, “clever” ways of working will need to be introduced until a vaccine is found to end the pandemic.

## Risk of horizontal transmission of SARS-CoV-2 and gynaecological surgery

SARS-CoV-2 is transmitted via the respiratory tract and the main risk of transmission to healthcare professionals remains highest at intubation and extubation during general anaesthesia (GA). These viral particles may also have the potential to spread as air droplets from released CO 2 during laparoscopic surgery or within surgical smoke produced from hysteroscopic, laparoscopic or open surgery. However, in contrast to aerosol generating procedures (AGPs) within the respiratory tract, the magnitude of SARS-CoV-2 viral transmission risks from airborne particles created during gynaecological surgery is likely to be low, although this remains uncertain.

Evidence suggests the risks of generating contaminated aerosols may potentially be lower with laparotomy when compared to laparoscopy (Li Cl et al., 2020a). Conversely, however, surgical smoke produced during laparoscopic surgery is collected in a confined space which, using methods previously described, can be safely evacuated from the abdomen (Mallick et al., 2020; European Society for Gynaecological Endoscopy, 2020). In open surgery, smoke dissipates into the theatre environment through larger wounds in a more uncontrolled manner and may potentially increase transmission rates.

Human-to-human transmission of the virus via direct contact, blood and faeces has also been described in the wider literature (Huang et al., 2020; Wang et al., 2020). SARS-CoV-2 viral RNA has been detected in faeces in up to 67% of COVID- 19 cases (Wang et al., 2020; Chen Y et al., 2020b). However, the live infectious particles have only been described in a significantly smaller number of cases (1-2%) (Wang et al., 2020). Therefore, while it is clear that SARS-CoV-2 can be transmitted by the faecal route, the low prevalence of live viral particles suggests that the potential transmission risk during surgery is low.

SARS-CoV-2 RNA viraemia is documented to have been detected in 97% of COVID-19 cases; however the actual viral RNA load is low, again suggesting a low risk of transmission from exposure to infected blood within escaping CO 2 aerosols or smoke (Rodriguez-Morales et al., 2020). The virus has not been identified in the urine or genital tract of female patients with COVID-19 (Chang et al., 2020; Chen Y et al., 2020b). Reassuringly this observation is supported by reports showing no evidence of vertical transmission in pregnant women suffering from COVID-19 (Fan et al., 2020; Chen H et al., 2020a; Chen Y et al., 2020c; Chen Y et al., 2020b).

There is conflicting evidence regarding the presence of SARS-CoV-2 in the semen of infected males. Li D. et al. (2020b) described the presence of SARS-CoV-2 in 16% of semen samples from a total of 38 men tested during the acute and recovery phase of COVID-19 . Conversely in a smaller study of 12 men in the acute and recovering phase of COVID- 19, Song et al. (2020) found no evidence of SARS- CoV-2 in semen samples . Coccolini et al. (2020) have reported on the first case of SARS-CoV-2 found in the peritoneal fluid of a COVID-19 patient. Again conversely, Ngaserin et al. (2020) reported the absence of SARS-CoV-2 in peritoneal fluid in an infected patient undergoing a laparoscopic appendicectomy.

## COVID-19 and surgical outcomes

Clinical outcomes appear to be worse in asymptomatic patients undergoing surgery with undetected COVID-19, although the evidence is limited (Lei et al., 2020). Specifically, the development of Adult Respiratory Distress Syndrome (ARDS) and need for ventilatory support, ITU admissions and overall mortality are higher. Surgery itself during the incubation period may worsen or accelerate subsequent disease progression. The added risks may be proportional to age, surgical complexity and patient co-morbidities; however these potential adverse outcomes must be taken on board when planning screening for COVID-19 and restoring non-essential surgery, and incorporated fully into patient counselling and consent.

## Reintroduction of services

Ideally before “normal” services can begin, the expectation would be to have an R-0 rate of less than one, thus decreasing the number of new infections. However, this will be different between regions and countries. The R (reproduction) rate is used to calculate how many new infections are caused by an earlier infection (Mahase, 2020). If the rate is below 1, the number of new infections is going down and conversely above 1 it is going up; however small differences in the R rate can lead to large changes in infection within the population. For example, if every infected person goes from infecting 2 people to 0.7 people over a 40 day period, the number of new cases would be one-sixth rather than 32 times at the higher figure.

In the United Kingdom (UK), for the reintroduction of routine services the government has set the following 5 targets to ensure that the National Health Service (NHS) can cope and have the ability to provide necessary critical care across the UK.

1. Seeing sustained and consistent fall in daily death rates
2. Be sure that the UK was beyond the peak of deaths
3. Falling infection rates
4. Adequate testing capacity
5. Personal protective equipment (PPE) availability to meet the future demand

The criteria for reintroduction of non-emergency surgery differs by region and by country. In the UK on 29 th April 2020, Simon Stevens, the NHS chief executive, wrote to all NHS trusts to prepare for the second phase of the NHS response to COVID- 19, (NHS England, 2020b) part of which was to reintroduce non-emergency surgery.

## Important points in reviving gynaecological surgery after COVID-19 Peak

As with many surgical specialities, over the last 2 decades with advances in technology and increasing expertise, minimal access surgery especially as day case procedures is the gold standard with well- documented health and economic benefits (Aarts et al., 2015; Hajenius et al., 2007). A balance between fixing the backlog of patients requiring surgery and maintaining high surgical standards needs to be fundamental to the reintroduction of surgery, as it would be unethical and wrong to lose these benefits post-pandemic as a consequence of patients having open surgery with low volume surgeons, just to expedite the large backlog of patients requiring surgery.

In the UK prior to the pandemic there were approximately 700,000 elective procedures a month and these cases will continue to accumulate as long as the pandemic and the diversion of healthcare services continue (Nuki, 2020). With a 3-month lockdown there will be an anticipated 2.1 million patients waiting for elective surgery. Banghu and colleagues, using a Bayesian beta-regression model, estimate that more than 28 million operations have been postponed worldwide during the 12 week pandemic peak, and that it would take a median of 45 weeks to clear the backlog (CovidSurg Collaborative, 2020).

## Patient prioritisation: scoring systems to ethically appoint patients

As the pandemic progressed and engulfed health care systems, organised stratification systems were used to identify the risks of the patient’s condition in relation to resources and the risk of surgery and time needed for recovery against a background of comorbidities (Goldman and Haber, 2020; Weber Lebrun et al., 2020). These systems can and should be adapted to the management of patients post-peak for the reintroduction of non-cancer gynaecological surgery.

After the peak of the pandemic, systems need to take into account that the patient was already booked for “elective” surgery, but the “elected” time was deferred due to a lack of resources coupled with the pandemic risks. Thus in the post-pandemic period a more suitable term to re-establish gynaecological operating would be “Medically Necessary Time Sensitive Surgery” (MeNTS) (Prachand et al., 2020). MeNTS is a system that uses a a five point scale of 1-5, with a higher value assigned for poorer perioperative patient outcome, increased risk of COVID-19 transmission to the health care team, and/or increased hospital resource utilisation. These are then anchored against objective and perceived variables again on a scale of 1-5 to produce an overall score. This score can then be used for the individual patient and be used by a committee of faculty members to evaluate overall suitability for surgery during the pandemic. This system described by Prachand et al. (2020) systematically integrates factors to facilitate decision-making and triage for surgery, and appropriately weighs individual patient risks with the ethical necessity of optimising public health concerns. The MeNTS scoring and triage process allows for offloading the emotional and ethical burden associated with having to make difficult decisions, weighing patient needs in the midst of resource scarcity and reducing the risk of transmission of COVID-19.

## Rationalisation of surgery

COVID-19 has been attributed to poorer perioperative outcomes, and the re-introduction of routine surgery will inevitably increase the potential transmission risks between patients and healthcare professions. Resources will need to be redistributed back to routine services and general hospital resource utilisation will increase. Given these factors, the patient’s need for surgery and the urgency of the planned procedure, taking into account these increased risks, will need to be classified according to:

1. The planned procedure
2. The disease requiring treatment and its natural history
3. The patient
  - Consideration of comorbidities
    - Particularly cardiopulmonary
    - Advanced age
    - Immunocompromised states

There is increasing evidence that the risks of and mortality from COVID-19 can be affected by socio- demographic factors. These include increasing age, obesity, male gender, occupation, and lower socio-economic groups (IFS, 2020, Simonnet et al., 2020). Data was also recently published by the UK Office for National Statistics (ONS), which clearly demonstrates that ethnic minorities are more likely to die from COVID-19 than Caucasians, with black females 4.3 times more likely to die than white ethnicity males and females (ONS, 2020). On the basis of these findings, it is important to individualise risk for each patient. However, it is well known that ethnic minorities in most societies suffer from disparity in healthcare provision, and gynaecology is not immune (Odejinmi et al., 2018). Thus, caution needs to be taken when individualising care and it must be ensured that ethnic minorities are not excluded from necessary surgery purely on the basis of race.

Guidance given by the Royal College of Surgeons of England and the British Association for Surgical Oncology suggest the following prioritisation criteria for surgery during the pandemic (NHS England 2020a).

- Priority level 1a: Emergency - operation needed within 24 hours
- Priority level 1b: Urgent - operation needed within 72 hours
- Priority level 2: Surgery that can be deferred for up to 4 weeks
- Priority level 3: Surgery that can be delayed for up to 3 months
- Priority level 4: Surgery that can be delayed for more than 3 months

The Royal College of Obstetricians and Gynaecologists have very recently produced guidance to aid the prioritisation of gynaecology- specific procedures into the above categories (Royal College of Obstetricians and Gynaecologists, 2020).

Another joint statement, which included the American Association of Gynecologic Laparoscopists (AAGL), produced guidance incorporating the above criteria specific to gynaecological procedures in relation to reintroduction of gynaecological and urogynaecological surgery (American Association of Gynecologic Laparoscopists, 2020b). Most of the medically necessary, time sensitive, major minimal access surgery in gynaecology will fall into the priority levels 3 and 4, depending on the presence or absence of comorbidities and the presence of anaemia.

**Figure 1:**
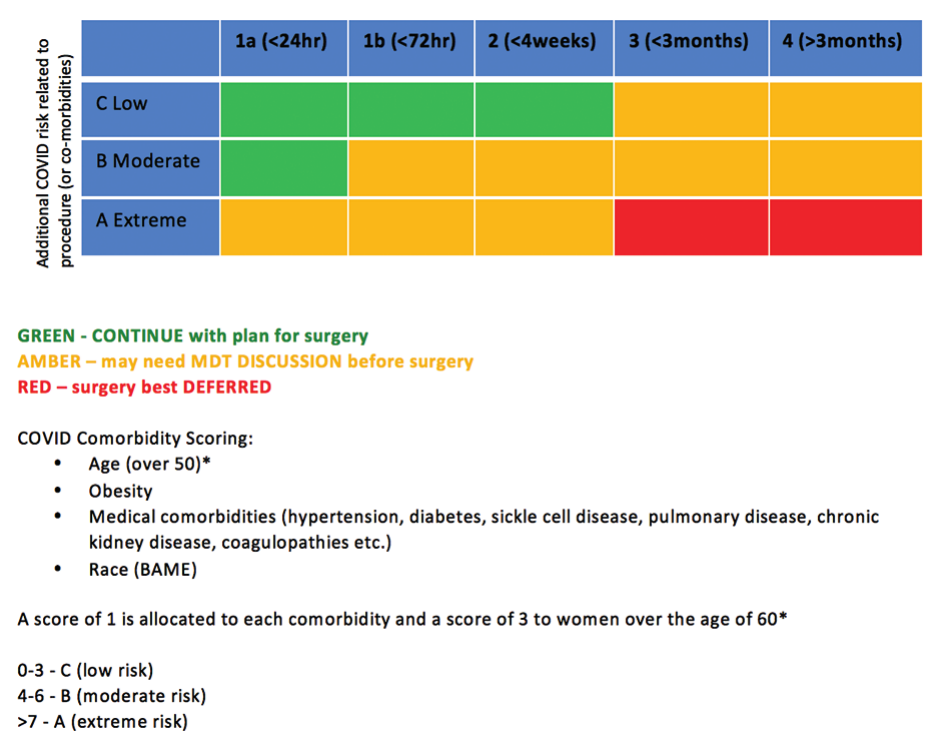
— A guide to prioiritsing patients awaiting benign gynaecolocial surgery

Figure 1 shows a modification of the grid that can be used to prioritise patients awaiting benign gynaecological surgery.

## Patient preparation and consent

After the peak of the pandemic, there will still be an ongoing risk to patients of contracting the virus as they come out of lockdown. Thus, the risk benefit analysis will need to be clear, weighing the risks of the morbidity associated with the patient’s benign condition against a background of possible medical comorbidities. There must also be up to date consent in line with the Montgomery ruling (Campbell, 2015), ensuring that surgery is still required. Non-surgical methods of treatment should be explored initially, provided they are a safe and effective alternative, in preference to surgery. All potential complications, particularly related to COVID-19, should be clearly discussed with the patient. Informed consent is key, and it is essential the patients understand the increased surgical risks in the context of COVID-19.

All patients awaiting surgery should be offered a further virtual consultation or face-to-face review, when clinically necessary, to discuss and document all the above. In patients who have experienced significant delays, repeat investigations may be necessary to re-evaluate pathologies such as adnexal masses, fibroids and endometrial polyps to ensure surgery is still appropriate.

## Screening of patients and COVID testing

### Elective surgery (day-case or inpatient)

All patients being admitted for elective surgery (day case and inpatient) should be screened using a screening questionnaire, taking into consideration any current symptoms, their contact history as well as a history of previous exposure and infection of family members.

All patients should also have their temperature recorded.

Patients should be advised to self-isolate for 14 days before their planned surgery in accordance with recent NHS England guidance (NHS England, 2020c). Furthermore, as it is unknown whether COVID-19 can be sexually transmitted, patients should be advised to also avoid sexual intercourse for the 14 days leading up to their procedure in view of the recent conflicting evidence of SARS- COV-2 found in semen (Li D. et al., 2020b; Song et al., 2020).

Further tests including lymphocyte count, ferritin, d-dimers, LDH and CT chest imaging are not generally required for benign gynaecological surgery, but may play a role in high risk patients and high-risk procedures such as cardiothoracic surgery.

COVID-19 specific testing that is currently available includes the polymerase chain reaction (PCR) antigen tests for SARS-CoV-2 for acute infections and antibody testing for evidence of past infection. Benefits of universal testing for routine cases include;

- a reduced risk of horizontal viral transmission
- reduced peri-operative complications from unrecognised SARS-CoV-2 infection

Emergency and urgent surgical cases should be tested for SARS-CoV-2 on admission, but surgery should not be delayed awaiting the result, unless time allows and without compromise to patient care. For patients who test negative, a further single re-test should be conducted between 5-7 days after admission (NHS England, 2020c).

The available reverse transcription- polymerase chain reaction (RT-PCR) antigen tests for SARS- CoV-2 are good at detecting COVID-19, but are of limited accuracy for excluding the virus with false negatives rates of between 30 and 50% (Alhazzani et al. 2020). Clinically, this means the test is very useful in detecting patients with COVID- 19, however due to the poor sensitivity in those who test negative, certain precautions may still be needed, such as ‘full’ PPE during surgery i.e. water repellent, long sleeved surgical gowns, eye and face protection, gloves and filtering face piece class 3 (FFP3). The role of IgG antibody testing in patients being admitted for gynaecological surgery is unknown and hence not currently recommended. All patients scheduled for elective surgery should be tested a maximum of 72 hours prior to their planned surgery, using standard oropharyngeal and nasal swabs, taking into account local turnaround times of testing (NHS England, 2020c). Following testing, all patients should be instructed to continue to self-isolate at home until the planned procedure date. All patients should undergo a screening questionnaire, temperature check and re-test on admission as this may be useful if they develop COVID symptoms post-surgery.

If a patient tests positive for SARS-CoV-2, surgery should be deferred for at least 14 days from the onset of symptoms and only when asymptomatic to avoid horizontal transmission.

Arrangements should be made for re-testing to ensure viral clearance in line with local policies.

Patients testing negative for SARS-CoV-2 but with positive screening questions at the time of testing, or subsequently on the day of admission for surgery, should be considered a suspected COVID- 19 case. Surgery should be deferred for 14 days and re-testing undertaken in line with local policies.

Patients testing negative for SARS-CoV-2 but with a temperature of greater than or equal to 37.3°C on the day of admission that is not attributable to the gynaecological condition necessitating surgery, should be considered a suspected COVID-19 case. Surgery should be deferred for 14 days and re- testing undertaken in line with local policies.

Patients testing negative for SARS-CoV-2 with negative screening questions and a normal temperature should be offered the minimally invasive approach if applicable and their procedure undertaken as planned. Full PPE should still be used, in open and laparoscopic procedures, and all other gynaecological procedures where diathermy is used, given the potential for aerosol transmission and high false negative rates of the test; however, this may differ between countries.

**Figure 2:**
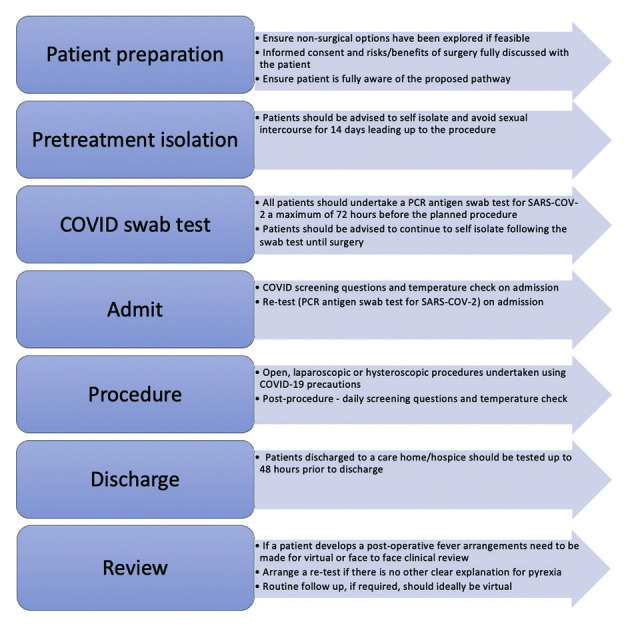
— Proposed Surgical Pathway

Following surgery patients should have daily screening questions and temperature checks until discharge. After discharge, a virtual follow-up consultation should be offered if clinically acceptable. If a patient develops a post-operative pyrexia, arrangements should be made for a virtual or face to face clinical review. SARS-CoV-2 re- testing should be undertaken if there is no other clear explanation for the pyrexia. All patients being discharged to a care home or a hospice should be tested up to 48 hours prior to discharge (NHS England, 2020c). A surgical pathway is proposed in Figure 2.

### Outpatient procedures

Patients attending for outpatient procedures such as hysteroscopy or manual vacuum aspiration (MVA) who screen positive on questioning (conducted according to local policies), should be considered a suspected COVID-19 case and undergo SARS- CoV-2 virology screening. Surgery should be deferred for 14 days and re-testing undertaken in line with local policies. This may not be clinically appropriate in certain circumstances, for example miscarriage, and should be assessed on a case-by-case basis.

Patients screening negative on questioning should undergo the procedure as planned. In procedures where diathermy is not used, full PPE is not required as SARS-CoV-2 has not been isolated in the female genital tract. Standard good practice and the avoidance of general contamination is recommended. In outpatient cases, where diathermy is utilised, given the small potential risk of transmission within surgical smoke, the use of full PPE should be at the clinician’s discretion and based on local guidance.

### The organisation

Organisations responsible for patient care need to endeavour that surgery remains safe; thus, a number of considerations are essential. Firstly, they need to ensure the re-assimilation of deployed staff to ensure units have adequate staffing to cope with the reintroduction of routine services. Many staff would have been redeployed to other departments within or outside their hospitals to help fight the COVID- 19 pandemic. The re-allocation of facilities is also essential, as many outpatient rooms, waiting areas and theatre spaces were utilised in the expansion of COVID-19 related patient care. It is also essential that there is adequate critical care capacity for high-risk elective patients before routine surgery is recommenced.

It is imperative that separate pathways are in place for both elective and non-elective patients, as well as COVID positive and negative patients, to protect both patients and staff. This may involve utilising separate floors, buildings or even hospitals and should specifically include separate theatres, recovery areas and ward facilities as well as separate staff groups. “COVID free” staff should be screened daily using an appropriate questionnaire as well as undergoing rapid PCR antigen testing if symptomatic. Regular swab testing to ensure asymptomatic COVID infection is not missed is also recommended. The role of IgG antibody testing in staff screening will become important in infection prevention and control when there is widespread access to reliable antibody testing for NHS staff and patients (NHS England, 2020c).

Infection control practices, including the use of PPE, should comply with local and national protocols. PPE including water repellent, long sleeved surgical gowns, eye and face protection, gloves and FFP3 respirators are recommended to be worn by medical and theatre personnel, during surgical procedures conducted under general anaesthesia (GA), to reduce the SARS-CoV-2 transmission risks. Finally, targets such as waiting list times must be re-evaluated and restored in a timely manner following lockdown.

### The theatre

Most standard operating theatres have a positive pressure environment relative to the surrounding air (e.g. in corridors and adjacent areas) to prevent the flow of air from less sterile areas into a more sterile one. However, this positive pressure environment makes the spread of aerosols faster, posing an increased airborne viral transmission risk. A negative pressure environment is ideal to reduce dissemination of virus and bacteria beyond the operating theatre, but such facilities are not widely available. A higher frequency of filtered air exchanges may help reduce viral load within an operating theatre (Wong et al., 2020). Standard positive pressure theatres typically allow 15-25 air changes per hour, whereas air may be changed more than 300 times per hour in operating theatres with laminar flow facilities; these are normally found in orthopaedic specific theatres. Furthermore, the risk of horizontal transmission of SARS-CoV-2 to healthcare staff can be reduced by ensuring only essential theatre personnel are present.

### The surgeon

During the COVID-19 pandemic many surgeons will not have operated for a significant period of time. The wider evidence highlights high volume surgeons have better outcomes (Mowat et al., 2016) and expert surgeons may need to work in groups to support those where required until surgical competencies are regained.

During lockdown many specialist organisations called for the suspension of elective work. Therefore surgical numbers required to maintain competencies in certain procedures will need to be reviewed, for example the British Society for Gynaecological Endoscopy (BSGE) requirements of 12 procedures of advanced laparoscopy for endometriosis per surgeon per year to retain BSGE centre accreditation.

Furthermore, with the redeployment of medical personnel, the UK General Medical Council (GMC) and other organisations have sent guidance surrounding the medico-legal implications of doing work outside a practitioner’s normal specialism. With the recommencement of normal services, the speciality medico-legal implications will resume.

## Potential burnout

The post-pandemic period will inevitably result in huge demands on the service and the ramping up of targets and increased surgeon workload, which has the potential for clinician burnout. It is imperative this is recognised, and support systems are put in place.

## Specific surgical considerations

### 

Surgical considerations, when operating during COVID-19 times have been well described (Mallick et al., 2020; British Society for Gynaecological Endoscopy, 2020; European Society for Gynaecological Endoscopy, 2020, American Association of Gynecologic Laparoscopists , 2020a). Specific considerations include (British Society for Gynaecological Endoscopy, 2020):

### Hysteroscopic surgery

1. Best practice should be followed for diagnostic/ operative hysteroscopy procedures to minimise the risk of general contamination from blood, urine, genital tract fluids and faeces.
2. Use mechanical instruments/tissue removal systems if feasible to minimise the generation of surgical smoke.
3. Where electrosurgery is used, facilitate the extraction of surgical smoke by using active suction connected to the outflow in a closed circuit.

### Laparoscopic surgery

1. Entry technique, instrument choice and port positioning should be according to the surgeon’s and hospital’s standard practice to minimise operative time and risk of complications.
2. Suction devices, smoke evacuation filters, retrieval devices and swabs should be used to prevent aerosol transmission, and remove smoke, aerosol and the CO_2_ pneumoperitoneum during procedures.
3. Only evacuate surgical smoke via the tap on ports when attached to a smoke evacuation filter and by direct suction using a vacuum suction unit.
4. Only evacuate the pneumoperitoneum via direct suction using a vacuum suction unit.
5. Avoid explosive dispersion of body fluids when removing trocars and retrieving specimens.
6. Special attention should be paid in cases of laparoscopic hysterectomy as there is a high risk of explosive dispersion of body fluid when the uterus is removed from the vagina. Swabs, suction and retrieval devices should be used to minimise droplet transmission and consideration should be given to performing an open hysterectomy, on a case by case basis.
7. When there is a risk of bowel surgery e.g. recto- vaginal endometriosis, adhesiolysis, the benefits of laparoscopic surgery should be weighed against the potential higher risk of SARS-CoV-2 transmission.

## Training implications

The impact of the COVID-19 pandemic on the education of surgical trainees has been documented; it has resulted in fewer training opportunities whilst the learning needs and requirements for the completion of training competencies have remained the same (Gallo and Trompetto, 2020; Potts, 2020). National bodies have emphasised that every effort is to be made to maintain the high standards of postgraduate medical education, despite the challenges of COVID (Health Education England, 2020). A consultant led service has been the mainstay during this pandemic, however as “normality” and routine gynaecological services resume, the training needs of the postgraduate trainees needs to be reviewed in detail and resumed as best possible. This may initially be participating in procedures, being involved in educational discussions even if not operating, and when operating they will need to be fully aware of operative principles as well as those surrounding PPE.

## Conclusion

The only certainty about this pandemic is that we will continue to learn more about this pathogen and the advice and guidance in combating this scourge will undoubtedly continue to change. As we enter the second phase of the response to the COVID- 19 pandemic with the flattening of the curve, it is important that organisations have robust pathways in place when reintroducing elective surgery, including the accurate triaging of patients and the urgency of their surgery, as well as testing and screening protocols for both patients and staff. The increased resource need and re-diversion of services as well as logistical considerations should be evaluated urgently to ensure facilities are able to provide COVID free theatre and recovery areas. Staff and patient safety remains paramount and clear information, as well the provision of adequate PPE, is essential. It is also important that organisations do not lose sight of the ethical considerations particularly with regards to procedure prioritisation and informed consent, especially when tackling this large backlog. Finally, it is imperative that staff and patients are aware of these new ways of working in order to prevent the added morbidity that could potentially result from surgical interventions.
